# Accuracy of axillary nodal ultrasound and ultrasound fine needle aspiration/core biopsy in the preoperative staging of patients with invasive breast cancer

**DOI:** 10.1186/bcr3764

**Published:** 2015-11-05

**Authors:** William Cheung, Suzanne McLenachan, Gauripriya Babu, Melanie Smith

**Affiliations:** 1 Breast Unit, Edinburgh, UK

## Introduction

Patients with invasive breast cancer undergo axillary ultrasound ± ultrasound fine needle aspiration (US-FNA)/core biopsy for preoperative staging depending on the ultrasound appearance. At our institution, abnormal axillary lymph node assessment includes: a cortical thickness >3 mm, focal or eccentric cortical thickening, nodal shape (spherical) and replaced appearance with loss of echogenic nodal hilum. Our aims were to evaluate the accuracy of preoperative US + US-FNA/core biopsy for detecting axillary metastatic disease.

## Methods

Excluding those patients who underwent neoadjuvant chemotherapy, we identified 120 patients with invasive breast cancer between January and December 2013, which yielded axillary node metastases on final surgical pathology. We performed a retrospective analysis of the clinical records and used descriptive statistics.

## Results

Preoperative US correctly identified 60/120 (50%) patients with axillary metastatic disease, 42/60 (70%) had subsequent true positive US biopsies. Of the cases where a biopsy was not performed, 88% (53/60) had one or two positive nodes confirmed after surgery and 12% (7/60) had at least three nodes. Thirty-four of 60 (57%) were from the symptomatic population. Of the total 21 false negative US biopsies from the 18 patients, 81% (17/21) were performed via FNA and 19% (4/21) via core biopsy. Eleven of 18 (61%) were from the symptomatic population. Twenty-nine of 42 (69%) true positive US biopsies were from the symptomatic population.

## Conclusion

The results highlight the need for a review of our biopsy criteria, which may result in a decrease in our biopsy threshold. An increase in the use of core biopsies may yield greater accuracy in correctly identifying axillary nodal disease.

